# Acquired Epidermoid Cysts of the Cauda Equina

**Published:** 2011-08-25

**Authors:** DA Nica, VED Strambu, T Roşca, D Cioti, R Copaciu, M Stroi, AV Ciurea, F Popa

**Affiliations:** * Neurosurgery Clinic, ‘Sf. Pantelimon’ Emergency Hospital, BucharestRomania; ** Surgery Clinic, ‘Sf. Pantelimon’ Emergency Hospital, BucharestRomania; *** Institute of Cerebro–Vascular Diseases and Microsurgery, BucharestRomania; **** First Clinic Neurosurgery, ‘Bagdasar–Arseni’ Emergency Hospital, BucharestRomania

**Keywords:** lumbar punctures, laminectomy, microsurgery

## Abstract

Intradural extramedullary epidermoid (EC) cysts are uncommon (0,2–1%). Acquired tumors appear more frequently as a late complication of lumbar punctures (40%). The authors present three cases of epidermoid cysts of the cauda equina which were surgically treated in their department during the past five years. All three had suffered lumbar punctures for rachianesthesia 6–9 years prior to their presentation. The patients' ages ranged between 19 and 31. Surgical treatment was deemed necessary because of the space–occupying nature of this slow–growing lesion, and this indication was supported by the MRI findings. Two–level laminectomy and microsurgical total tumor ablation were performed in all three cases. There were no postoperative complications.

## Introduction

Epidermoid cysts (EC) are benign tumors which take the form of nests of cutaneous tissues that migrate improperly along the line of neurocutaneous ontogenetic differentiation during neural tube closure [1,2,3]. They can be identified by their lining of stratified squamous epithelium. They are uncommon, solitary lesions, accounting for 0.5–1% of all spinal tumors. They rarely suffer malignant transformations [4] in the context of prolonged chronic inflammation at the site of the EC, leading to the development of a squamous cell carcinoma.

Due to the slow growth of compact keratin, there is a slow onset of symptoms caused by the space–occupying EC to the detriment of neighbouring nervous tissue [3] (in our case, the apex of the medullary cone and the roots of the *cauda equina*). Being dermoid tumors, EC can appear in the spinal cord, subdurally, extramedullarly [5], in the middle ear, the brainstem, at the pontocerebellar angle, or inside the brain or the calvaria.

Macroscopically, extramedullary subarachnoid epidermoid cysts have an ellipsoidal shape and are surrounded by a whitish and poorly vascularised capsule, which can adhere to the neighbouring elements (pia mater, arachnoid, the roots of the cauda equina, fillum terminale and the medullary cone and epicone). Their strong subcapsular wall is generated when the local squamous epithelium spirals into pearly–white strata of brittle keratin on the inside of the subcapsule [6, 7].

There are two main types of EC: congenital and iatrogenic [8]. Congenital EC are formed when the existing neural tube, comprising the neural line and the ectoblast engaged in neural and cutaneous origins, fails to correctly close during weeks 3–5 of intrauterine life, trapping cutaneous cells within it. [9] These cells can serve as the origin of an EC [10,11,12].

Congenital EC can be suspected in case of occult or obvious defects in the closure of the rachis, in the absence of local trauma and in symptomatic patients under the age of 20. The iatrogenic hypothesis comes into play when patients report having suffered a lumbar puncture [13] for rachianesthesia or meningitis, or when the patients are over 20 years old and lack rachial closure abnormalities. Iatrogenic EC have also been reported in literature after the incidental formation of a skin pocket during suturing [2,14,15,16].

CT scans depict EC as homogenous encapsulated masses with variable small calcifications inside their capsules. In medullary–CT and cysternography, the contrast medium surrounds the cyst and gives the impression of total arrest on sagittal reconstructions.

On MRI images the tumor appears slightly hyperintense in T_2_ [2].

We shall proceed to the presentation of the three cases of EC, operated on in Sf. Pantelimon Hospital, Clinic of Neurosurgery, within a six–year interval (November 1999 – June 2005).

## Case Reports

### Case 1–T.A. 21–year–old female

The patient had been suffering from lumbalgia, which gave in at rest for approximately three years. This evolved into bilateral, predominantly left, lombocruralgias and ‘saddle’ paresthesias during the last two months. During the final three weeks of these months, the patient also complained of a retention–type sphincter malfunction. Her medical history was notable for an appendectomy with rachianesthesia six years prior to the current presentation. ‘Saddle’ paresthesias and bilateral L3 hypoesthesia and urinary retention due to sphincter malfunction were demonstrated by the neurological exam. 

Paraclinically, frontal and lateral lumbar spine X–rays did not show Elberst–Dicke changes (distancing of the vertebral pedicles), scalloping or dysraphism. Lumbar spine MRI showed an intradural and extramedullar oval tumoral formation of 3/1.7/2.7 cm at the site of the L_1_ and L_2_ vertebral bodies, with an isosignal with marrow in T_1_ and hypersignal in T_2_, situated posteriorly to the spinal cord.

Surgery consisted of L_1_ and L_2_ laminectomy, longitudinal sectioning of the dura matter under x 2.5 magnification, with temporary suspension and the complete ablation of an ellipsoidal tumoral formation, with a long axis of 3.2 cm and a small one of 1.7 cm. The formation had a whitish, relatively transparent capsule, extremely poorly vascularised and contained a pearly–white, friable core surrounded by concentric strata. The right anterolateral portion of the tumor adhered rather laxly to two of the equinal roots, due to the presence of arachnoiditis. These adherences were separated under magnification.

Histopathologically (microscopically):There were no postoperative complications, which generally take the form of chemical meningitis, hemorrhage or CSF fistulae. 90% of the patient's neurological complaints were gone immediately after the surgery, and the rest of her complaints faded within three months.

### Case 2–C.C. 31 year–old female

The patient presented with hemi–caudal syndrome with a slow, progressive onset of approximately 6 months, consisting of pain in the right leg, on the anterior aspect of the thigh and knee, with paresthesias in the same territory and retention–type sphincter malfunction–listed in their order of appearance. The patient's medical history was positive for one lumbar puncture performed for rachianesthesia prior to an appendectomy. 

Neurologically, the patient adopted an antalgic decubitus, Lasegue's sign was positive at 50 degrees of angulation, algic hyperesthesia was demonstrated at the L_2_ and L_3_ territories on the right and the retention of urine was objectified.

Paraclinically, no abnormalities were seen on frontal and plane spinal X–Rays. Lumbar myellography described an absence of contrast medium extending caudocranially from L_2_ to T_12_. Native CT showed a well–circumscribed mass inside the spinal canal with a density of 42 UH (isodense), surrounded by the remains of the opaque post–myellography contrast medium with a relatively cystic aspect. The mass was strictly median and did not compress adjacent roots. It blended in with the surrounding structures and seemed to extend cranially to the T_12_ vertebral body. Lumbar spine MRI showed a similar tumoral formation placed behind the medullary cone, displacing it anteriorly.

Surgery was performed under x 2.5 magnification and consisted of total ablation of a 3.7/2.7/2.2 cm tumor. The extension towards T_12_ which was unclearly visualized on the CT was in fact an arachnoid cyst which adhered to the superior pole of the tumor.

The histopathological exam described a cystic formation surrounded by keratinized pluristratified squamous epithelium with a core of keratin plates.

No meningeal reactions were noted postoperatively. The patient's recovery was spectacular, and her symptoms disappeared within approximately two weeks.

### Case 3–D.N.S. 19 year–old female

The patient presented with ‘balancing’ cruralgias in the L_3_ area of both thighs for approximately two months, muscular weakness in the same territory and retention–type urinary sphincter malfunction for approximately two weeks.

The patient had a lumbar puncture for rachianesthesia for appendectomy nine years prior to our neurosurgical consultation.

The objective neurological exam showed a limping patient with predominantly rhizomyellic paraparesis. Mingazzini's sign was positive bilaterally and there was bilateral hypoesthesia in the L_3_ territory. Urinary sphincter malfunction with urinary retention was objectified.

Paraclinically, the frontal and lateral spine X–Rays were devoid of any abnormality. MRI of the lumbar spine showed an oval mass of 4.5/2 cm at the site of the L_2_ and L_3_ vertebral bodies, with a hyposignal in T_1_ and hypersignal in T_2_. The mass was situated posterolaterally to the left, displacing the anterior and right lateral medullary cones, with a total extramedular arrest on myellography.

Surgery followed a similar protocol to those mentioned above. The tumor was considerably larger in vivo (6.5/3/2.5 cm), and a small portion of the inflamed arachnoid could not be completely removed as it adhered closely to one of the roots of the *cauda equina*. 

There were no postoperative complications.

## Discussions

Intradural extramedulary iatrogenic epidermoid cysts (EC) are more frequent in the lumbar region, due to the preferred site for lumbar punctures, and form approximately 40% of all spinal EC [17,5]. Performing a lumbar puncture without a rigid guide needle can inadvertently bring epidermic cells into the subarachnoid region. These benign masses present as intradural space–occupying lesions which compress the medullary cone and the roots of the cauda equina. They have a slow growth process [12] lasting for many years, and symptoms tend to appear several years after the incriminated spinal taps and progress at a similar drawn–out rate. EC occupies the intradural space, becoming ellipsoidal with a vertical long axis as a result [18]. 

**Figure 1:**
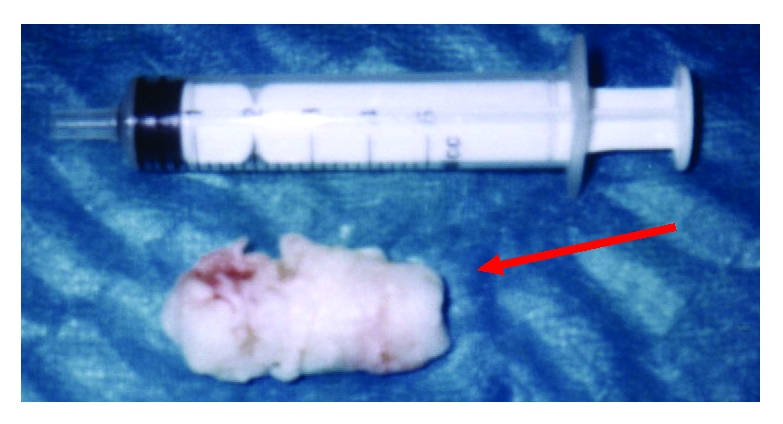
The dimension of the epidermoid cyst with its elipsoidal aspect

In the three cases we have previously described, we observed that the bigger the size of the tumors the  greater the time passed between the lumbar puncture and surgery:

- Case 1: 6 years and 3.2/1.7/1.7 cm
- Case 2: 7 years and 3.7/2.7/2.2 cm
- Case 3: 9 years and 6.5/3/2.5 cm (Figure 1)

The slowly–evolving neurological symptoms associated with this condition tend to be unsystematic and generally gravitate towards the ‘superior caudal syndrome’, sometimes with added elements that suggest the compression of the medullary cone. If a cyst were to rupture in the preoperative phase, the symptoms would appear rather abruptly and would be caused by granulomatous meningitis appearing as a defensive immune reaction to the presence of keratin cells in the CSF [19,20]. 

**Figure 2:**
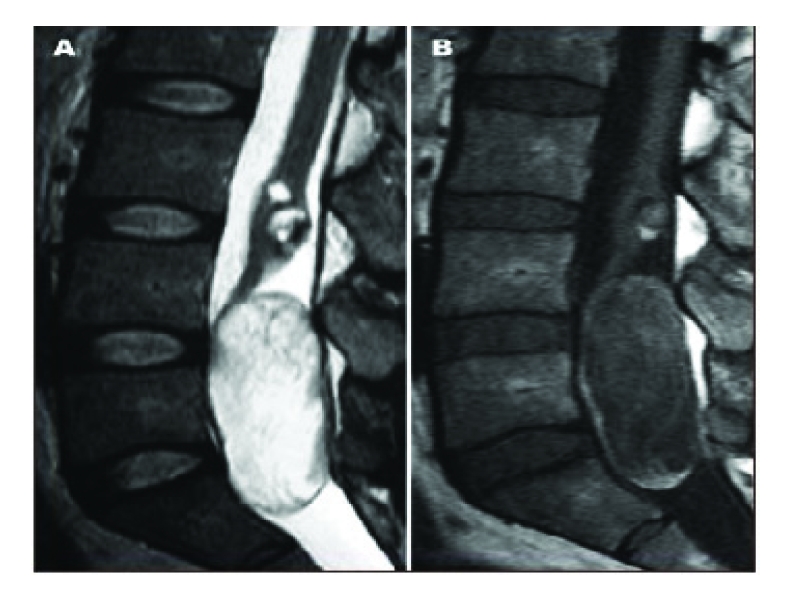
MRI image of cauda equina epidermoid cyst: note anterior displacement of the medullarycone

Imaging studies demonstrate that the MRI, particularly FLAIR sequences, is essential in diagnosing this condition and evaluating the need for surgical intervention in EC [21,22]. Thus, the masses are hypodense in T_1_ sequences and hyperdense in T_2_ sequences (Figure 2). 

The difference between the predicted dimensions of the lesion on MRI and their sizes in vivo may be due to the decompression of the tumor after laminectomy, the opening of the dura and the spontaneous evacuation of pressurized CSF. 

**Figure 3:**
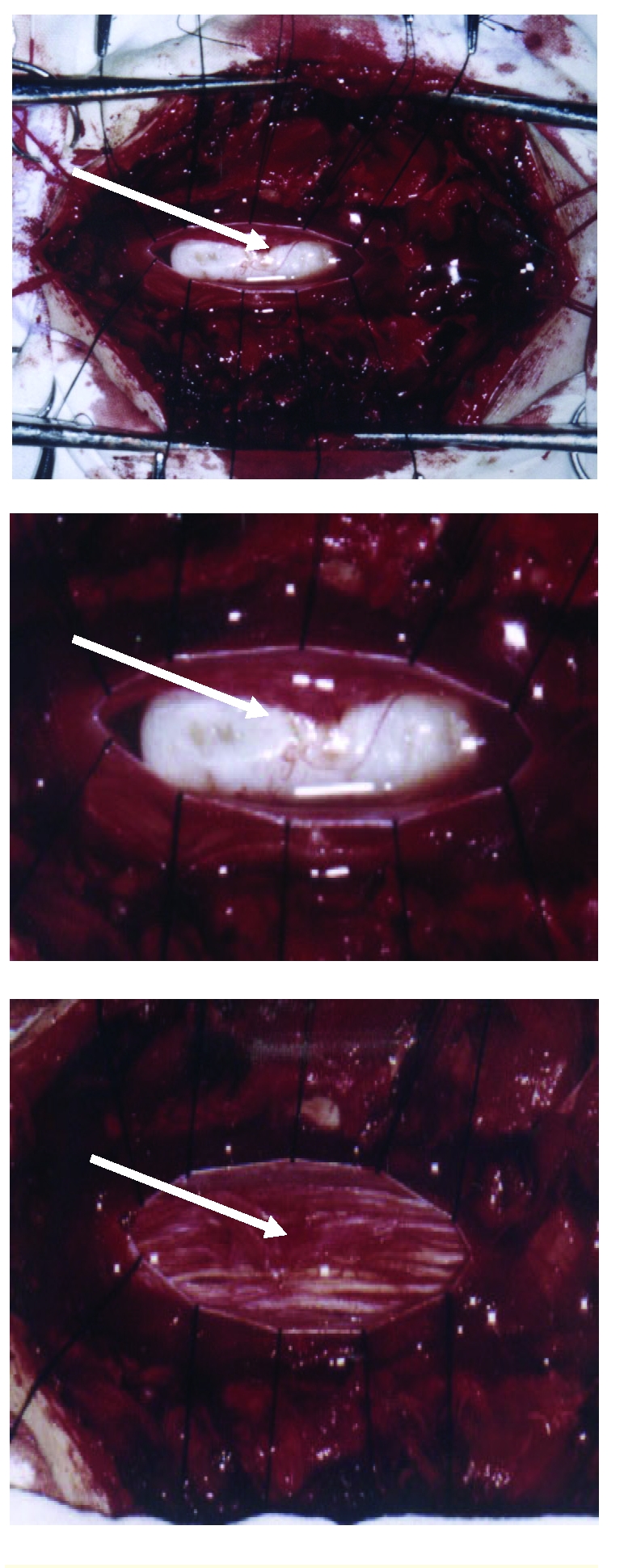
a) Aspect of intradural tumor; b) Dura was opened. The tumor in situ. (Photo without magnification); c) Aspect of the tumor in situ with 2.5 x magnification; d) The aspect of cauda equina after cyst removal.

All three cases were surgically treated under general anesthesia with orotracheal intubation due to the duration of the surgery (median = 4.5 hours). Staged laminectomy on two levels, with or without slighting of the supra– and subjacent plates, was radiologically oriented towards the tumor (C–arm, Seremobile) and revealed the tumor prior to the incision of the dura (Figure 3). 

After a median incision of the dura extending at least 1 cm superiorly and inferiorly to the tumor and temporary suspension, the patient is placed in the Trendelenburg position and two gauze pads are placed above and below the tumor to limit the loss of CSF and prevent further dissemination of the cyst's cells. 

After exposing the tumor, the separation of its adherences to the pia mater, arachnoid, medullary cone and the roots of the cauda equina and the subsequent ablation of the tumor were performed under x 2.5 magnification with microsurgical instruments. 

Total ablation is never possible (not even in the case of small EC) because of the aforementioned adherences. The danger of placing traction of nervous and vascular elements and thus causing microthromboses and postoperative neurological deficits was avoided by debulking the tumor [22]. 

In order to avoid simultaneous dissemination of the components of the cyst, continuous  irrigation and aspiration of the intradural space between the two gauze pads was performed. The ablation was total in all three of the cases, although a small portion of inflamed arachnoid was left in place in Case 3, as it adhered closely to a surrounding nervous root.

The region was closed by using 3.0 Ford interlocking sutures, and anatomic parietorraphy was performed after epidural drainage. There were no early or late intraoperative or postoperative complications (intraoperative bleeding, meningitis, CSF fistulae or wound infection).

Dexamethasone (8mg/8h IV) was administered in all three cases preoperatively, Solu–medrol was given intraoperatively and Dexamethasone was continued postoperatively at every 12 hours. Cases 2 and 3 also received Cerebrolysin (10 mL x 2/ day). The patients were mobilized on the second postoperative day with the help of a kinetotherapist. Almost 90% of their symptoms receded in the early postoperative period, and the rest disappeared within three months from surgery at most.

The three surgeries were performed in 1999, June 2004 and June 2005 and there have been no reported recurrences thus far. These recurrences usually appear between five and nine years after surgery [22,23].

## Conclusions

Lumbar puncture may be associated with EC, but more patients are needed in order to support this hypothesis. The presence of at least one spinal tap in the patient's history [13,17,23], age of onset of symptoms between 19 and 31, the direct correlation between the size of the tumor and the time it has evolved, in and the presence of radiologic dysraphism, may indicate a iatrogenic cause.

Differences between the MRI–predicted and real size of the tumor may be due to the decompression of the tumor. During staged laminectomy, the opening of the dura and the release of pressurized CSF were noted as preliminary observations resulted from these 3 interventons.

The ideal goal of the surgeries we performed was total microsurgical ablation of the EC, paying great attention to avoiding contact between the interior of the tumor and CSF (secondary meningitis).
